# Implication of Contactins in Demyelinating Pathologies

**DOI:** 10.3390/life11010051

**Published:** 2021-01-13

**Authors:** Ilias Kalafatakis, Maria Savvaki, Theodora Velona, Domna Karagogeos

**Affiliations:** Laboratory of Neuroscience, Department of Basic Science, University of Crete Medical School and IMBB FORTH, Nikolaou Plastira 100, 70013 Vassilika Vouton, Greece; ilias_kalafatakis@imbb.forth.gr (I.K.); msavaki@imbb.forth.gr (M.S.); theodora_velona@imbb.forth.gr (T.V.)

**Keywords:** contactins, nodes of Ranvier, demyelination

## Abstract

Demyelinating pathologies comprise of a variety of conditions where either central or peripheral myelin is attacked, resulting in white matter lesions and neurodegeneration. Myelinated axons are organized into molecularly distinct domains, and this segregation is crucial for their proper function. These defined domains are differentially affected at the different stages of demyelination as well as at the lesion and perilesion sites. Among the main players in myelinated axon organization are proteins of the contactin (CNTN) group of the immunoglobulin superfamily (IgSF) of cell adhesion molecules, namely Contactin-1 and Contactin-2 (CNTN1, CNTN2). The two contactins perform their functions through intermolecular interactions, which are crucial for myelinated axon integrity and functionality. In this review, we focus on the implication of these two molecules as well as their interactors in demyelinating pathologies in humans. At first, we describe the organization and function of myelinated axons in the central (CNS) and the peripheral (PNS) nervous system, further analyzing the role of CNTN1 and CNTN2 as well as their interactors in myelination. In the last section, studies showing the correlation of the two contactins with demyelinating pathologies are reviewed, highlighting the importance of these recognition molecules in shaping the function of the nervous system in multiple ways.

## 1. The Role of Contactins in the Organization of Myelinated Axons

**Figure 1:**
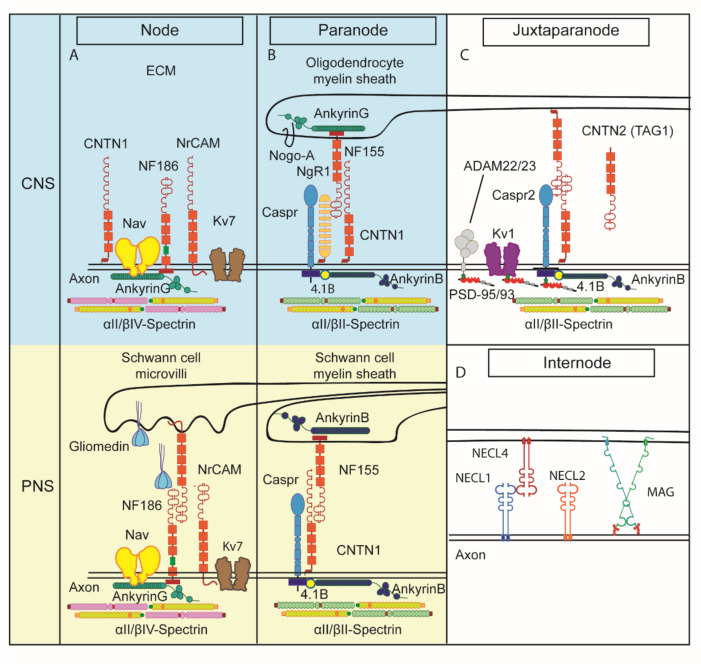
Molecular structure of the perinodal areas. Schematic representation of the four specialized domains encountered along a myelinated axon. (**A**) Nodes, (**B**) paranodes, (**C**) juxtaparanodes and (**D**) internodes. Nodes and paranodes differ in molecular composition between the central nervous system (CNS) and the peripheral nervous system (PNS). In the blue background are depicted the CNS, while in yellow, the PNS node and paranode. The most notable difference between the two systems is the presence of Schwann microvilli in the PNS node, while the CNS node is mostly surrounded by extracellular matrix (ECM) components and astrocyte processes (not shown). Nodes contain clustered Na_v_ channels while K_v_ channels are accumulated in the juxtaparanodes (JXPs). Each domain is enriched with cell adhesion molecules (CAMs) and other proteins that are important for the clustering of the channels (details in text), while the paranodal junction complex separates the two clusters from each other. Contactins are present in these complexes, with Contactin-1 (CNTN1) being specifically enriched in the paranode and the CNS node, while Contactin-2 (CNTN2) contributes to the JXP complex. Further away in the internode, axo-glial contacts and signaling are mediated by nectin-like proteins and myelin-associated glycoprotein (MAG). Cytoskeletal elements like ankyrins and spectrins differ between the nodes and the rest of the areas.

Myelinated axons are characterized by exquisite architectural and functional specificity. They are endowed with the task of rapid signal propagation, which they effectively accomplish by segregating large adhesion protein complexes and ion channels along the axon, thus generating distinct functional and morphological domains, namely the node of Ranvier, paranode (PNJ), juxtaparanode (JXP) and internode (Figure 1) [1,2,3,4,5,6,7,8,9,10,11,12,13,14,15,16].

### 1.1. Node of Ranvier

The nodes, named after French neurologist Louis Ranvier who first reported them in 1871 [17], are the myelin-free axonal intervals and form the anatomical basis of saltatory conduction in both the CNS and the PNS. In these ~1 μm long axonal segments that are organized with regular periodicity, ion flow occurs resulting in the propagation of action potentials along the axon. Two processes, the ensheathment of axons by myelin-forming glial cells and the clustering of Na_v_ channels, key elements of action potential generation and propagation in the myelin-free nodes, are essential for fast conduction. While in unmyelinated axons, the Na_v_ channels are distributed along the axon, in myelinated axons, they are clustered in the nodes [18,19]. The molecular composition of the nodes has been intensively studied, permitting a comprehensive view of the key molecules present in the axolemma ([20,21,22] most recently reviewed in [8]). The nodal complex consists of a multitude of voltage-gated sodium channels (Na_v_; so far, four alpha subunits, five beta subunits have been detected in the nodes) and some subtypes of voltage-gated potassium channels (K_v_ 7.2–7.3) among scaffolding proteins and the underlying actin cytoskeletal elements ankyrin-G and βIV-spectrin. Figure 1A depicts our current view of the main components of the CNS and PNS nodes. In the PNS, Schwann cell projections, namely microvilli, closely oppose the axolemma and secrete gliomedin, a glycoprotein with extracellular matrix features which in turn binds to the nodal cell adhesion protein neurofascin186 (Nfasc186) [23]. Nfasc186 is a partner of Na_v_ resulting in the clustering of the latter and further links the nodal complex to the underlying cytoskeleton via interaction with scaffolding proteins [24,25,26].

Schwann cells control the assembly of nodes of Ranvier by two different mechanisms, which operate simultaneously during development, the clustering and the restriction mechanism. The clustering mechanism requires the establishment of axo-glial contact between Schwann cells microvilli and the axolemma and is mediated by gliomedin, NrCAM and NF186 as described above. In contrast, the restriction mechanism does not require these three nodal CAMs but depends on axo-glia interaction at the paranodes, which is mediated by Caspr, CNTN1 and NF155, as detailed in the section below. These two mechanisms are sufficient for the assembly of mature PNS nodes [23].

Even though the PNS and CNS nodes of Ranvier do not have many changes in their molecular composition, the mechanisms regarding their formation differ. In contrast to the PNS, in the CNS, there are three complementary mechanisms participating in nodal assembly: ECM-induced cell-- adhesion molecule clustering, paranodal barrier formation (see below) and scaffolding molecules anchoring the nodal complex to the actin cytoskeleton [27].

In contrast to Schwann cells, oligodendrocytes do not contact nodes directly and do not express gliomedin. Recent data showed that an oligodendrocyte conditioned medium on hippocampal neurons induces prenodes associated with an increased conduction velocity [28]. In addition, the authors identified CNTN1 combined with RPTP/phosphacan or tenascin-R as the proteins that are able to induce clusters of nodal proteins along hippocampal GABAergic axons [29]. If CNTN1 is removed from the conditioned medium or if conditioned medium from OCM from *Cntn1^−/−^* mice are used, reduced clustering activity is evident, which is restored by the addition of soluble CNTN1 [29]. There are other glia-derived ECM proteins such as chondroitin sulfate proteoglycans, tenascin-R, Bral1 that form complexes with axonal CAMs such as Nfasc186, NrCAM, CNTN1 and the b-subunits of sodium channels [27]. CNS nodes of Ranvier can also be assembled through intrinsic neuronal mechanisms directed by axonal scaffolding proteins such as ankyrinG. This protein binds several membrane-spanning axonodal proteins through its multiple ANK repeats and connects them to the neuronal actin cytoskeleton, playing an important role in the formation of the macromolecular complex at the nodes [30].

Nfasc186 is also expressed in the CNS, involved in the mechanism of Na_v_ clustering at nodes [26]. Another isoform of neuronal neurofascin, Nfasc140, as well as the immunoglobulin superfamily (IgSF) glycoprotein NrCAM are also present in the node of both CNS and PNS, although their function is not established [31,32,33]. Finally, in the CNS, CNTN1 is shown to be present in the nodes, while in the PNS, its nodal presence manifests transiently only during remyelination. CNTN1 can directly interact with sodium channels in transfected cells [34].

### 1.2. Paranode

The nodes are flanked by the largest intercellular adhesion complexes found in vertebrates, the paranodal junctions (PNJ, Figure 1B; [35]). They represent the main regions of interaction between the overlying myelin and the axolemma and are visualized in the electron microscope as septate junctions. PNJs operate as diffusion barriers for the segregation of sodium and potassium voltage-gated ion channels found at nodes and juxtaparanodes, respectively, and are thought to block the passage of nodal currents into the internode [7]. PNJs assemble early during the myelination process to drive the conjunction of glial processes. In mice, the lack of intact PNJs leads to severe defects in saltatory conduction and early death [36]. The paranodal barrier formed through direct axoglial contacts established at the PNJ also participates in the assembly of CNS nodes of Ranvier [37]. The reconstitution of paranodes by glial expression of Nfasc155 is sufficient to rescue Na_v_ channel clustering.

The paranodal complex involves glial Nfasc155, axonal Contactin-associated protein 1 (Caspr, a neurexin family member), as well as axonal CNTN1 ([36,38,39,40,41,42,43], most recently reviewed in [44]). The absence of any one of these three molecules, as shown in loss of function studies, leads to paranodal disruption with progressive loss of axo-glial interactions, defective ion channel segregation and impaired nerve conduction [26,36,41,45]. The phenotype of *Cntn1*-deficient mice in both the PNS and CNS is a clear disruption of the PNJs, loss of paranodal Caspr and mislocalized voltage-gated potassium channels, which are normally present in the juxtaparanodes [36,46]. Another important component of this region is the axonal cytoskeleton. In recent years, paranodal cytoskeleton, consisting of αII spectrin, βII spectrin, ankyrin B and protein 4.1B, has been shown to be indispensable for the integrity of the region [47,48] and plays a role in glial processes extension in the CNS [49]. Progress on the mechanisms by which the paranodal complex and its underlying cytoskeleton participate in node formation has allowed the elucidation of several aspects of the nodal assembly.

### 1.3. Juxtaparanode

The juxtaparanodes (JXPs) are flanking the PNJs comprising part of the internodal compact myelin. Their organization and maintenance depend on the combination of two distinct processes, the lateral diffusion barrier created by the PN domain and the formation of the unique juxtaparanodal membrane complex and its linkage to the cytoskeleton (Figure 1C). The complex that organizes this region consists of CNTN2 (previously named axonin-1/TAG-1) present on the glial and axonal membranes as well as the contactin-associated protein 2 (neurexin protein Caspr2) and the Shaker-type voltage-gated potassium channels (VGKCs) on the axon, a hallmark of this region [50,51,52,53,54,55,56,57,58]. The phenotype of *Cntn2^−/−^* mice shows that axonal Caspr2 does not accumulate at the JXPs while the VGKCs is severely disrupted in the CNS and PNS with significant impairments in learning and memory [54,56,57]. The juxtaparanodal complex is connected to the actin-spectrin cytoskeleton mainly via protein 4.1B [59,60,61,62,63]. Previous work has shown that the paranodal cytoskeleton, consisting of αII spectrin, βII spectrin, ankyrin B and protein 4.1B is indispensable for the structure not only of the PNJs but also of adjacent regions such as the JXPs [47,48]. In addition to the three-tier JXP complex, additional molecules are preferentially localized in this region, albeit not affecting VGKC clustering, such as the metalloproteinases ADAM-22 and 23 [64,65,66,67,68].

The nature of the JXP axo-glial interaction has been demonstrated to some extent with the use of *Cntn2^−/−^* mice. Glial CNTN2 associates *in trans*, homophilically with axonal CNTN2, which interacts *in cis* with axonal Caspr2 [54]. Thus, along with 4.1B, these three proteins anchor the VGKCs under the juxtaparanodal myelin. Whether this tripartite interaction affects VGKC function still remains unclear. VGKCs may not contribute to AP conduction significantly. However, they may have a role in maintaining the internodal resting potential or preventing rapid re-excitation [50,51].

### 1.4. Internode

The largest domain of the myelinated fiber is the internode, which represents the area of compact myelin between adjacent nodes of Ranvier (Figure 1D). The members of the Nectin-like family of adhesion molecules (Necl proteins, also known as SynCAM or Cadm), a small group of Ig cell adhesion molecules, were identified as key regulators of the internodal domain organization. These proteins are linked to the cytoskeleton via a FERM-binding domain and a class II PDZ sequence found on their C-terminus, which is capable of 4.1 protein binding [69,70]. A recent study in the mouse and zebrafish have shown that paranodal and internodal (namely myelin-associated glycoprotein, MAG) adhesion molecules work synergistically to regulate CNS myelin growth [71].

## 2. Implication of Perinodal Contactins and Their Interactors in Demyelination

**Figure 2:**
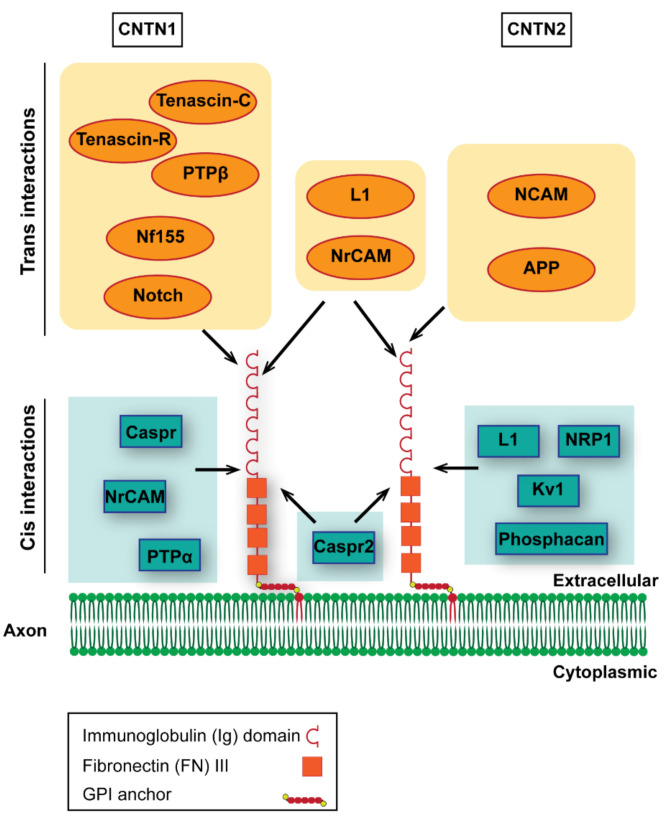
Contactins 1 and 2 and their interactors. Contactins 1 and 2 are the members of the contactin subfamily of the Ig superfamily involved in the organization myelinated axon areas. Their overall structure is very similar, with each containing six Ig type C and four FNIII domains. They are linked to the axon and/or myelin membrane through a glycosylphosphatidylinositol (GPI) anchor. Apart from their role in myelin domain organization, these Contactins are important for axon outgrowth and fasciculation through heterophilic interactions with partners that belong to several families of molecules. Trans interactors of CNTN1 involve members of the L1 family of IgCAMs, like neurofascin 186, NrCAM and L1, and ECM components, like tenascin-R, tenascin C and RPTPβ/phosphacan. Finally, CNTN1 interacts in trans with Notch receptors. Cis interactions involve again the L1 CAM NrCAM, ECM component protein tyrosine phosphatase (PTPa) and the molecule Caspr/paranodin. Contactin2 interacts in trans with L1 Ig family NrCAM and L1 as well as IgCAM NCAM. CNTN2 also binds amyloid precursor protein (APP) in trans. Cis interactions involve K_v_1 and Caspr2 in the juxtaparanode of the Ranvier node, the IgCAM L1, Nrp1, through which it modulates Sema3A signaling and the ECM component phosphacan. Depending on the presence of interactors as well as the cellular context, Contactins may exert different functions.

Contactins comprise a subfamily of the immunoglobulin superfamily of adhesion molecules. They are characterized by a glycosylphosphatidylinositol (GPI) anchor; therefore, they are released from the plasma membrane. All members have at least one isoform comprising of six Ig-C2 domains and four fibronectin 3 (FNIII)-like domains. They share a 45–65% protein sequence identity [72,73]. In mice, there are 6 Contactins, expressed mainly in the nervous system displaying a distinct and partially overlapping pattern ([72] and reviewed in [15,16,74]). Of these, CNTN1 and 2 are the ones implicated in myelinated axon organization, so we will mainly focus on these two members (Figure 2). All Contactins studied have been shown to be involved in shaping nervous system development, and some have been correlated with known human neurological disorders as addressed in Section 4. As detailed in this section, CNTN1 antibodies have been implicated in peripheral demyelinating neuropathies, while CNTN2 antibodies have been detected in a small subset of multiple sclerosos (MS) patients.

### 2.1. Contactin-1 and CIDP

CNTN1, as well as other proteins detected in the paranodal complex of myelinated fibers, have been implicated in chronic inflammatory demyelinating polyradiculoneuropathy (CIDP). CIDP is the most common form of chronic inflammatory neuropathies, which comprise a group of rare diseases associated with motor and sensory deficits with varying disabilities [75]. CIDP is affected by environmental and genetic factors [76,77] and results from an immune-mediated response primarily against the PNS [78]. Clinically, CIDP is described as a chronic and insidious neurological disorder associated with relapses and re-currencies lasting more than 8 weeks. It is characterized by symmetric paresthesias, weakness, and sensory dysfunction in extremities. CIDP is also characterized by areflexia, cranial nerve involvement, autonomic symptoms, neuropathic pain and demyelination [79]. The pathogenesis of CIDP involves inflammatory cell infiltration and segmental demyelination, especially in the paranodal region, resulting in defective nodal segments with sheaths of thinner myelin [78].

Antibodies against paranodal antigens have been implicated in CIDP [80,81]. Anti-CNTN1 antibodies are found in samples of CIDP patients [82,83,84]. These antibodies were mainly IgG4, which is an isotype that does not efficiently promote inflammation, while their pathogenic effect becomes clear from the paranodal destruction observed in myelinated fibers from skin biopsies of anti-CNTN1-positive CIDP patients [75,81,85,86,87]. Previous studies showed that anti-CNTN1 IgG4 antibodies disrupt the CNTN1-CASPR1-NF155 complex, thus promoting the disruption of the PNJ without involving inflammation [85,88]. Alternatively, the clinical phenotypes of CIDP could be a result of the impaired, autoantibody-mediated function of CNTN1 in myelination [89,90]. The presence of autoantibodies against the CNTN1 or CNTN1/CASPR complex in CIDP patients suggests that these could play a pathogenic role, and for this reason, they could serve as diagnostic biomarkers in these patients [88,90]. IgG4 anti-NF155 antibodies have also been detected in CIDP patients. Sural nerve biopsies from CIDP patients that were positive for these antibodies showed paranodal demyelination without signs of inflammation as well as the loss of septate-like junctions with interposition of cellular processes between the paranodal loops and the axolemma. These observations suggest that IgG4 anti-NF-155 antibodies can also disrupt the NF155-CASPR1-CNTN1 complex located at paranodes [26,89,91,92]. Finally, anti-CASPR1 antibodies have been associated with inflammatory neuropathies. More specifically, there were patients with CIDP and Guillain–Barré syndrome (GBS) that were positive for anti-CASPR1 antibodies and negative for anti-CNTN1 or NF155 antibodies. Guillain–Barré syndrome (GBS) is a paralytic demyelinating immune-mediated neuropathy. Analysis of myelinated fibers in skin biopsies revealed the paranodal disruption, while these patients also had intense neuropathic pain [75]. Neuropathic pain was the primary result of anti-Caspr antibody binding in these patients suggesting that the paranode is a primary target of the pathology of inflammatory neuropathies [85,93].

Patients with sera antibodies against paranodal proteins often display aggressive onset of the disease, while they may also be diagnosed with GBS [90]. Anti-CNTN1 antibodies are associated with aggressive disease, denervation at onset and poor response to intravenous immunoglobulin (IVIg), which is one of the most well-known immune therapies. Patients with anti-NF155 antibodies show distal weakness and tremor, while they also respond poorly to IVIg treatment [75]. As previously mentioned, paranodal autoantibodies are almost all of the IgG4 isotype. It is worth mentioning that the prevalence of autoimmune diseases known to be mediated by IgG4 autoantibodies rapidly increases in recent years, making urgent the early detection of paranodal autoantibodies for the prediction of the clinical phenotype and, more important, for the selection of the therapeutic approach [75,87].

### 2.2. Contactin-2 and MS

CNTN2 and its interactors, as part of the JXP complex, have been implicated in inflammatory neuropathies, while their effects are more prominent in MS, as indicated by animal and human studies. More specifically, single nucleotide polymorphisms (SNPs) related to the Cntn2 gene could predict responsiveness to intravenous immunoglobulin (IVIG) treatment in patients with CIDP [94], but its presence was not correlated with clinical characteristics of the disease [95]. Furthermore, Caspr2 autoantibodies have been associated with cases of pediatric Guillain-Barre syndrome [96].

MS is the most common demyelinating disorder, comprising a devastating autoimmune neurodegenerative disease of the CNS, characterized by myelin loss, varying degrees of axonal pathology and progressive neurological dysfunction. It affects young adults between 15 and 55 years of age and has a severe impact on patients’ quality of life with a prevalence that varies markedly geographically [97]. A series of studies in human MS tissue and mouse models of MS indicate that the juxtaparanode is severely disrupted during demyelination and is restored during remyelination as part of this late process [12,13,98]. The PNJs are shown to be disrupted, followed by the JXPs in MS tissue [12,13,99,100]. Autoantibodies recognizing CNTN2 and Caspr2 have been identified in patients with MS, indicating that T-cell response is directed against the juxtaparanode during the progression of MS [101]. The presence of CNTN2 autoantibodies in the serum of MS patients could not differentiate between early occurrence and stages of MS (clinically isolated syndromes, relapsing–remitting, secondary-progressive, and primary-progressive) or different MRI profiles [102]. However, the data presented in [101] showed inflammation of gray-matter blood vessels, and thus, the authors tested a “two-hit” model in EAE rats by administering anti-MOG antibodies after the administration of contactin 2-specific T cells. They noticed widespread demyelination in white but also gray matter. Based on their results, they proposed that immune-mediated inflammation against CNTN2 on gray-matter endothelial cells opens the BBB, allowing demyelinating agents (such as antibodies to myelin proteins) to enter the gray matter. This model predicts that CNTN2 may present itself as a sensitive marker of gray-matter disease [103].

Immunohistochemical analysis of human MS tissue showed that the JXP organization is defective not only in the active demyelinating lesion in both the gray and white matter but also in the normal-appearing white matter (NAWM). More specifically, post-mortem histological examination of MS brains revealed diffuse distribution mostly of VGKCs, but also of CNTN2 and Caspr2 in demyelinating plaques, while in remyelinating lesions, VGKCs and Caspr2 were re-clustered [12,13]. mRNA and protein levels of juxtaparanodal molecules were also affected, as shown with material from the lesion area [13].

### 2.3. Contactins as Biomarkers?

CNTN1 and CNTN2 protein levels in the CSF have been found to vary in different stages of MS. Levels of both proteins were found reduced in established relapsing-remitting type MS (RRMS) patients in comparison to control individuals, which was not the case for primary progressing MS (PPMS) patients [104]. On the contrary, strong enrichment of grey matter components, among which CNTN2, was found in the CSF of early MS patients (first episode or clinically isolated syndrome-CIS) when compared to patients and control individuals [105]. An increase of CNTN2 in the CSF early on in MS could indicate axonal dysfunction and/or loss on the first appearance of the disease, which may lead to the release of axonal elements into the CSF. Indeed, CNTN2 levels reflect the presence of already known axonal degeneration markers, such as neurofilaments, in the CSF of patients with clinically isolated MS [104]. On the other hand, the reduction of CNTN1 and CNTN2 levels in established RRMS patients possibly reflects a mechanism aiming to compensate for nodal disorganization in early MS. Finally, the presence of juxtaparanodal components in the CSF may be used to predict disease progression after a first demyelinating episode. For example, CNTN2 predicts very well subsequent brain atrophy in CIS patients [104]. Furthermore, in children with acquired demyelinating syndrome (ADS), CNTN2, neurofascin, NrCAM and ADAM22 levels were increased in the CSF of children who later developed MS versus ones with monophasic ADS [106].

In summary, CNTN1 and 2 are implicated in human disease, albeit in a different manner. CNTN1 antibodies are associated with CIDP mainly, while autoantibodies to CNTN2 are detected in a small fraction of MS patients. In human MS tissue, both paranodal and juxtaparanodal, as evidenced by the expression of CNTN1 and 2, respectively, are disorganized. At the same time, protein levels of these proteins are detected in the CSF, possibly indicating axonal damage correlating with the disease. A Table (Table 1) summarizing CNTN1 and 2 antibodies and demyelinating pathologies is included.

## 3. Perinodal Contactins and Their Interactors in Animal Models of Demyelination

Even though at this moment there is no animal model to recapitulate all aspects and stages of MS, several mouse models have been developed that allow the study of different features and stages of the disease (inflammatory, autoimmune and demyelination-remyelination processes) [107,108,109]. In these models, demyelination is brought about through immune system stimulation, local or systemic toxin introduction, genetically encoded toxin expression or viral transduction. During the last two decades, studies on two of these models, the experimental autoimmune encephalomyelitis (EAE) and the cuprizone demyelination model, have provided insight into the effects of demyelination in the organization of the node of Ranvier and the contribution of contactins and their interactors in the perinodal area to the pathogenesis of MS and other demyelinating conditions.

### 3.1. Contactins and EAE

The EAE model provides the closest simulation of MS progression, in which demyelination is considered to be the result of a primary attack of the CNS by the immune system. In this model, immunization against myelin is achieved either actively, by injecting purified myelin-specific proteins or peptides, the most used being MOG, myelin basic protein (MBP) and proteolipid protein (PLP), or passively through the administration of already activated T cells. The antigens or T cells are injected together with complete Freund’s adjuvant, which activates the peripheral immune system, toll-like receptors and Th1 immune response and increases blood–brain barrier (BBB) permeability. Bordetella pertussis toxin injections may also be implemented to further stimulate the immune system and enhance BBB permeabilization [108,110,111].

In MOG-induced EAE in mice, demyelination causes elongation of Caspr-positive paranodes and a decrease in their number [98]. Furthermore, the VGKCs are dramatically reduced during the peak of demyelination, while the remaining ones have diffused in the internode, and Caspr2 and CNTN2 clustering is also disturbed. Partial remyelination in this model restored a great number of PNJ and partial restoration of JXP. Protein levels of all three components are reduced in the spinal cord during full demyelination and remained reduced in partial remyelination conditions [98].

In MOG-EAE rats, injection of monoclonal anti-pan-NF antibodies, which bind specifically neurofascin (NF186), aggravate the disease and axonal damage, even though there is no additive effect in demyelination and inflammation [112]. Furthermore, in a passive model of EAE, adoptive transfer (direct injection of ex vivo activated immune cells) of CNTN2-specific CD4+ T cells induced gray matter inflammation in rats [101] to a bigger extent than in the MOG-EAE model, suggesting a specific role for autoimmunity against CNTN2 in gray matter inflammation and damage.

Demyelinating optic neuritis (ON) is studied in the context of the EAE model of autoimmune inflammatory demyelinating disorders in the CNS, certain variations of which are more relevant to ON than others, depending on the species and strain [113]. Apart from optic nerve demyelination, ON is characterized by infiltration of autoantibodies against various targets. Nodes and paranodes start to be disorganized before the initiation of demyelination, as Nav and Caspr-positive clusters appear to be elongated compared to control mice [114].

Autoantibodies against Caspr1 and CNTN1 located in the paranodes of the optic nerve but also in the ribbon synapses of the unmyelinated retina are detected in the EAE model even before the onset of optic nerve demyelination [115]. These mice present initial defects in synaptic vesicle cycling and visual perturbation, suggesting an early implication of synapses in the pathogenesis of optic neuritis. Consistent with this idea, Caspr-CNTN1 interaction is disrupted by rotenone-mediated inhibition of the mitochondrial complex in the visual pathway of mice. This disorganization leads to demyelination and visual loss, a phenotype that is rescued through the replacement of complex I by a substitute NADH dehydrogenase [116].

In summary, the EAE model has been extensively used to understand or simulate certain MS manifestations mainly centered around the inflammatory aspects of the pathology. In this model, paranodal (CNTN1-expressing) and juxtaparanodal (CNTN2-expressing) disruptions have been recognized. In addition, autoantibodies to both proteins have been detected before the onset of ON demyelination.

### 3.2. Contactins and the Cuprizone Model

The cuprizone toxic demyelination mouse model is a systemic toxin administration model in which the inclusion of the copper chelator cuprizone in the diet of adult mice induces oligodendrocyte toxicity and leads to reproducible damage in CNS myelin. It has been proposed that cuprizone-induced toxicity is caused through inhibition of mitochondrial complex IV [117], but the exact mechanism remains unclear. In contrast to the EAE model, in the cuprizone model, the BBB remains intact and T cells are not implicated. Demyelination is observed three weeks after the onset of toxin administration and complete demyelination is seen five to six weeks into the treatment. Removal of cuprizone from the diet frequently leads to complete remyelination within four weeks [107]. This model has permitted the study of molecular and cellular mechanisms of demyelination and especially remyelination, with the goal to devise therapeutic strategies permitting to block the former and enhance the latter [118].

Demyelination induced by cuprizone administration causes a similar phenotype with EAE-induced demyelination with regard to PNJ components. On the other hand, while JXP complex clustering is lost in demyelinating conditions, complete remyelination occurring after removal of the toxin from food is sufficient for restoration of clustering of these components, unlike in the EAE model [98].

Lack of CNTN2 in a complete knockout mouse model leads to defects in oligodendrocyte maturation both in vivo and in vitro [67]. Furthermore, in a combination of cuprizone treatment with in vivo ablation of CNTN2, K_v_ channels are able to re-cluster in the juxtaparanode during remyelination [67]. This re-clustering is accompanied by an increase in the callosal compound action potentials (CAP) responses in remyelinating CNTN2-deficient mice. Given that Caspr2 is also absent from the juxtaparanode in these mice while ADAM23 and 4.1B are present, one can hypothesize that ADAM23, K_v,_ and 4.1B form a complex in the absence of Caspr2 and CNTN2 in remyelinating conditions.

In conclusion, the cuprizone model has proven a very useful experimental model of demyelinating diseases as it may mimic certain key features such as remyelination, among others. It is particularly amenable to pharmacological investigations. CNTN2 as a marker of JXPs has been explored, and both CNTN2-dependent and well as independent mechanisms of JXP assembly during remyelination have been revealed.

Finally, in a focal lysolecithin-induced demyelination model in rats, apotransferrin-induced remyelination was characterized by overexpression of CNTN1, which may induce oligodendroglial maturation through Notch activation, thus promoting remyelination [119].

## 4. Other Functions of Perinodal CNTN1 and 2

### 4.1. Contactin-1

CNTN1 has been detected in the retina, spinal cord, cerebral cortex, hippocampus and cerebellum, as well as oligodendrocytes [6,120,121,122]. In addition to its role in organizing the perinodal domains, described in Section 1, CNTN1 also plays important roles in the hippocampus, augmenting synaptic plasticity, neurogenesis, and memory in adult mice [123,124,125]. CNTN1 overexpression leads to inhibition of neurogenesis, which is associated with Notch pathway activation [126,127,128,129]. It is also associated with the promotion of oligodendrocyte differentiation again through Notch pathway activation [130,131]. Both Hes-1 and Deltex-1 dependent pathways were found to be activated by CNTN1-Notch interactions. The former pathway is responsible for early CNTN1 inhibitory effects on the neuronal lineage [132], while the latter one is responsible for its effects in promoting oligodendrocyte differentiation [133], suggesting that CNTN1 interactions with the Notch receptors differentially contribute to the modulation of neural developmental events. Another important regulator of oligodendrocyte maturation and differentiation implicating CNTN1 is its interaction with PTPRZ (protein tyrosine phosphatase, receptor-type, Z polypeptide 1), a protein primarily expressed by OPCs, astrocytes, and mature oligodendrocytes [134,135,136,137].

Apart from its aforementioned roles, CNTN1 also plays a crucial role in both myelination [46] and axonal regeneration [138] by modulating these processes via axo-glia interactions. CNTN1 also affects myelination through its participation in a tripartite complex of CNTN1-fyn-PTPα, promoting the interaction of PTPα (receptor protein tyrosine phosphatase) and fyn, a non-receptor tyrosine kinase [137,139]. Fyn is an important regulator of myelination as it participates in a number of pathways involved in oligodendrocyte development, acting in OPCs, OLs and initiating CNS myelination (reviewed in [140]).

### 4.2. Contactin-2

CNTN2 (or TAG-1, as originally named in rodents and axonin-1 in the chick) is a glycoprotein expressed in various subsets of neuronal cells predominantly, but not exclusively on their axons (as detailed in [141,142,143] and reviewed in [14,15,16]. CNTN2 exists as a neuronal as well as a glial isoform with distinct functions and is found highly enriched in lipid rafts in an environment of sphingolipids [55,56,58,142,144,145]. A great number of studies have established a role for the rodent and chick homologs in axon outgrowth, pathfinding, fasciculation and tangential migrations ([142,146,147,148,149,150,151,152,153,154], reviewed in [14,15,16]). Although the human ortholog (TAX-1) is not directly involved in human disease, it is mapped in the 1q32.1 locus [155], a chromosomal region linked to several malignant gliomas, the Usher syndrome type II related to retinitis pigmentosa and the Van der Woude syndrome of craniofacial abnormalities [156,157,158].

Other than its interactions with other IgSF members, most notably L1 and NrCAM, CNTN2 has been recognized as a ligand for APP to negatively impact neurogenesis [159]. CNTN2 regulates the endocytic trafficking and signaling of another important complex, the sema3A receptor complex [160]. The expression of neuronal but not glial CNTN2 negatively regulates axon regeneration in the injured adult optic nerve. In this process, a novel CNTN2 interactor, the truncated form of tropomyosin-related kinase receptor-B (TrkB), has been implicated (Savvaki et al. in press).

As summarized in the sections above, CNTN2 influences myelination due to its pivotal role in organizing the juxtaparanodes. Additionally, it is involved in the development of oligodendrocytes and can transiently affect the expression levels of myelin and myelin-regulating genes. Under demyelinating conditions, CNTN2 does not affect the OLC number or the extent of remyelination. However, a CNTN2-independent and possibly compensatory mechanism has been described during remyelination leading to increased conduction ([67] and the section on the juxtaparanodes above).

## 5. Concluding Paragraph

Data that we have reviewed in this work show that CNTN1 and CNTN 2 play diverse and important roles in myelinated fiber organization by positioning essential elements of neuronal function such as voltage-gated sodium and potassium channels. Moreover, it is clear that they influence the trafficking and positioning of many membrane components into specific domains of the axon. By facilitating the clustering of membrane proteins in distinct axonal domains via homophilic and heterophilic interactions, they are able to modulate responses from the extracellular milieu to the cytoskeleton.

Our deep knowledge of the basic biology of axo-glial interactions is a prerequisite in understanding the pathophysiology of myelin-related pathologies. Despite the great progress, a clear picture of how disruption of axonal domains leads to myelin pathology is still missing. The elucidation of key pathways that maintain axo-glial integrity may also provide clues as to novel therapeutic targets.

## Figures and Tables

**Table 1:** Table summarizing contactin and contactin interactors-related demyelination antibodies and diseases.

Contactin and Contactin Interactors-Related Demyelination Antibodies	Associated Demyelinating Diseases
Anti-CNTN1	CIDP
Anti-NF155	CIDP
Anti-CASPR1	GBS, CIDP
Anti-CNTN2	MS
Anti-CASPR2	GBS, MS
Anti-NFASC	MS
Anti-NrCAM	MS
Anti-ADAM22	MS

CIDP: chronic inflammatory demyelinating polyneuropathy, GBS: Guillain–Barré syndrome, MS; multiple sclerosis.
